# Venomous Snakes Reveal Ecological and Phylogenetic Factors Influencing Variation in Gut and Oral Microbiomes

**DOI:** 10.3389/fmicb.2021.657754

**Published:** 2021-03-26

**Authors:** Sierra N. Smith, Timothy J. Colston, Cameron D. Siler

**Affiliations:** ^1^Sam Noble Oklahoma Museum of Natural History and Department of Biology, University of Oklahoma, Norman, OK, United States; ^2^Department of Biology, University of Florida, Gainesville, FL, United States

**Keywords:** 16S rRNA, host ecology, mouth microbiome, Philippines, venomous snakes, gut microbiome

## Abstract

The gastrointestinal tract (GIT) of vertebrates contains a series of organs beginning with the mouth and ending with the anus or cloacal opening. Each organ represents a unique environment for resident microorganisms. Due to their simple digestive anatomy, snakes are good models for studying microbiome variation along the GIT. Cloacal sampling captures the majority of the microbial diversity found in the GIT of snakes—yet little is known about the oral microbiota of snakes. Most research on the snake mouth and gut microbiota are limited to studies of a single species or captive-bred individuals. It therefore remains unclear how a host’s life history, diet, or evolutionary history correlate with differences in the microbial composition within the mouths and guts of wild snakes. We sampled the mouth and gut microbial communities from three species of Asian venomous snakes and utilized 16S rRNA microbial inventories to test if host phylogenetic and ecological differences correlate with distinct microbial compositions within the two body sites. These species occupy three disparate habitat types: marine, semi-arboreal, and arboreal, our results suggest that the diversity of snake mouth and gut microbial communities correlate with differences in both host ecology and phylogeny.

## Introduction

The organs within the gastrointestinal tract (GIT) of vertebrates (mouth, stomach, colon, cloaca, etc.) harbor microbiomes that serve fundamental roles in a variety of processes that benefit their animal hosts, including digestion, immunity, and nutrient acquisition (Colston and Jackson, 2016; Varela et al., 2018; Arizza et al., 2019; Qin et al., 2019). As such, studies of microbiomes are essential to understanding host health, and can also be utilized to address interesting questions across broad fields in evolutionary biology, from processes of coevolution and adaptation to the evolution of antibiotic resistance (Hird, 2017; Ul-Hasan et al., 2019). To date, most vertebrate microbiome research has focused on humans and other mammals (Colston and Jackson, 2016; Hird, 2017; Qin et al., 2019); however, increased availability and utilization of culture-independent techniques, such as next-generation sequencing technologies and 16S ribosomal RNA (rRNA) microbial inventories, has expanded our knowledge of the diversity, structure, and potential functional capabilities of these symbiotic bacterial communities (Bletz et al., 2017; Medina et al., 2017). Furthermore, such techniques have allowed for microbiome studies across an increasingly wide taxonomic diversity of vertebrate organisms, including birds (Hird et al., 2015; Waite and Taylor, 2015), fishes (Clements et al., 2014; Givens et al., 2015; Sullam et al., 2015; Gajardo et al., 2016), amphibians (Bletz et al., 2016, 2017; Bird et al., 2018; Varela et al., 2018; Rebollar and Harris, 2019), and reptiles (Hong et al., 2011; Keenan et al., 2013; Colston et al., 2015; Hyde et al., 2016; Ren et al., 2016; Allender et al., 2018; Arizza et al., 2019; Tang et al., 2019). Despite these advances, large gaps remain in our understanding of the non-mammalian vertebrate microbiome, especially among wild reptiles (Colston and Jackson, 2016; Hird, 2017; Krishnankutty et al., 2018; Qin et al., 2019; Tang et al., 2019).

Squamate reptiles (snakes, lizards, and amphisbaenids) possess the ability to persist in a wide variety of habitats, occurring on every continent but Antarctica (Vitt et al., 2003; Vitt and Caldwell, 2013). These reptiles display a vast diversity of life history traits, particularly in dietary habits and reproductive modes (Shine and Bonnet, 2000; Vitt and Caldwell, 2013). They have also played an important role in addressing many higher-level questions in ecology and evolutionary biology across numerous fields of study that include using venom to study evolutionary key innovations (Casewell et al., 2013; Sunagar et al., 2016; Calvete, 2017) and studies of phenotypic evolution (Wagner et al., 2018; Watanabe et al., 2019), reproductive mode (Blackburn, 2006; Pyron and Burbrink, 2014), and adaptive radiation (Losos and Miles, 2002). Despite such a rich history of foundational research on the group, microbiome evolution among squamate reptiles remains poorly understood. Previous work has determined that different reptilian body sites along the GIT possess distinct microbiomes (Keenan et al., 2013; Colston et al., 2015; Hyde et al., 2016; Tang et al., 2019); however, few studies to date have investigated the diversity and composition of the squamate reptile mouth microbiome (Shek et al., 2009; Goldstein et al., 2013; Hyde et al., 2016; Krishnankutty et al., 2018). Additionally, most studies of the reptilian gut (endogenous, cloacal) microbiomes are limited to comparisons between individuals of the same species (Keenan et al., 2013; Colston et al., 2015; McLaughlin et al., 2015; Hyde et al., 2016; Allender et al., 2018; Arizza et al., 2019; Tang et al., 2019), with few studies investigating how the gut microbiome differs between taxa (Hong et al., 2011; Ren et al., 2016; Qin et al., 2019; Zhang et al., 2019). By comparing the microbiomes of reptiles that have different habitat preferences, diets, and ecologies, we can begin to understand the relationship between evolutionary factors and the diversity and composition of host-associated microbial communities.

By far one of the most successful and charismatic radiations of squamates are snakes (Squamata: Serpentes), with more than 3,840 species recognized currently (Uetz et al., 2020) across all major biomes on the planet, including freshwater and marine environments, and even southern regions of the tundra (Vitt and Caldwell, 2013). Represented by this global vertebrate radiation is an incredible diversity of ecologies and life histories, including the evolution of a wide spectrum of dietary preferences and adaptations (Vitt et al., 2003; Colston et al., 2010; Vitt and Caldwell, 2013; Tang et al., 2019). Interestingly, such variation in diet preferences and the degree of specialization has evolved despite the presence of a rather simple digestive anatomy, and for this reason snakes are widely used as ideal systems for studying digestive physiology (Secor and Diamond, 1998; Castoe et al., 2013). Unfortunately, we continue to have a limited understanding of the composition, diversity, and functional capabilities of snake microbiomes, particularly gut and mouth microbial communities (Krishnankutty et al., 2018)—a critical component to understanding the important roles host-specific microbiomes may have played in snake adaptive evolution. Currently, the culture-independent snake gut microbiome literature consists of a few studies that describe the diversity present at different segments of the snake gastrointestinal tract (excluding mouth; Hill et al., 2008; Colston et al., 2015; McLaughlin et al., 2015; Tang et al., 2019), one study investigating the composition of gut microbiota in captive pythons (again, excluding mouth; Costello et al., 2010), one study comparing small and large intestinal bacteria among three species of snakes (Qin et al., 2019), and one comparative study of bacterial communities sequenced from fecal samples from four species of farmed snakes in China (Zhang et al., 2019). Such a general paucity of data on snake host-associated microbiomes extends to the burgeoning studies of the venom-microbiome, which aims to describe the presence and diversity of venom-associated microbial communities to address questions of how microorganisms colonize and inhabit venom glands (McFall-Ngai, 2014; Ul-Hasan et al., 2019). For example, the oral cavity of snakes can harbor an extensive diversity of bacteria, including potentially pathogenic groups that may cause post-bite infection (Shek et al., 2009; Lam et al., 2011; Krishnankutty et al., 2018). Such host-microbe interactions that occur in the venom microenvironment and the oral cavity of snakes also remain largely understudied (Ul-Hasan et al., 2019). Until more comparative and baseline data on host-specific microbiomes among snakes becomes available, our understanding of how host ecology, venom system dynamics, and evolutionary history correlate with, and are impacted by, these microbial communities remains incomplete.

In this study, we present novel comparisons of host microbiome diversity and composition among three venomous snake species from the Philippines in Southeast Asia, each of which possesses a distinct ecology: (1) the blue-lipped sea krait (Family Elapidae: *Laticauda laticaudata*) is a neurotoxic, marine species (Leviton et al., 2014); (2) the Philippine pit viper (Family Viperidae: *Trimeresurus flavomaculatus*) is a hemotoxic, strictly-arboreal, terrestrial species (Sugihara et al., 1983; Nikai et al., 1985; Debono et al., 2019); and (3) the mangrove snake (Family Colubridae: *Boiga dendrophila*) is a semi-arboreal, terrestrial species that possesses a bird-specific toxin in its venom referred to as denmotoxin (Pawlak et al., 2006; Pawlak and Kini, 2008). These key differences, and the general availability of these three species in the field, made them ideal for investigating the influence and interaction of host ecology and evolutionary history (phylogenetic relatedness) on gut and mouth microbiome structure and diversity. *Laticauda laticaudata* is an amphibious marine snake in the family Elapidae that is widely distributed across Southeast Asia and comes to shore only to rest and lay eggs (Leviton et al., 2014). This species can be observed hiding in crevices on coral reefs and they feed primarily on eels (Dabruzzi et al., 2012). The two terrestrial species, *T*. *flavomaculatus* and *B*. *dendrophila*, are common snakes found throughout large regions of the Philippines (Siler et al., 2011; Brown et al., 2013). *Trimeresurus flavomaculatus* is an ovoviviparous pit viper in the family Viperidae that prefers more arboreal microhabitats, often preying on frogs and small mammals (Leviton, 1962; Brown et al., 2013; Leviton et al., 2014). In comparison, *Boiga dendrophila* is an oviparous mangrove snake in the family Colubridae that prefers more shrub- and ground-level microhabitats (i.e., semi-arboreal) and feeds primarily on birds (Brown et al., 2013).

Herein, we provide an assessment and summary of microbial diversity and composition of the mouth and gut microbiomes among these three species of wild venomous snakes to identify patterns associated with host ecology (e.g., habitat preferences and specializations, etc.) and host species differences. The results contribute to a nascent body of literature on wild reptile endogenous microbiomes and specifically expands our knowledge on snake mouth and gut microbiomes, with which researchers can begin to address broader questions in evolutionary biology and digestive physiology as they apply to host-microbe interactions.

## Materials and Methods

### Sample Collection

Fieldwork was conducted from May 27 to June 4, 2018 on two islands of the Babuyan Island Group—Calayan and Camiguin Norte. Cloacal swabs are an effective proxy for sampling gut microbial diversity in snakes (Colston et al., 2015), therefore we used cloacal swabs to sample gut microbial diversity in this study. Gut microbiome samples were collected by inserting a sterile swab (MWE, Corsham, United Kingdom) into the cloaca of the individual for approximately 3–4 s, twirling the swab 2–4 times. We collected mouth microbiome samples by swabbing the tongue, teeth, and roof of the snake’s mouth 4–5 times with sterile swabs (Puritan Medical Products, Guilford, ME, United States). We swabbed 22 adult individuals representing the three focal species: *Laticauda laticaudata* (*N* = 7 [four males, three individuals not sexed], SVL 680–855 mm), *Trimeresurus flavomaculatus* (*N* = 7 [four females, three individuals not sexed], SVL 308–900 mm), and *Boiga dendrophila* (*N* = 8 [four males, four females], SVL 814–1,395 mm) (Supplementary Table S1). All swabs were preserved and stored in DNA/RNA Shield (Zymo Research Products, Irvine, CA, United States) at the time of collection at ambient temperature in field conditions until returned to the United States (10–14 days) where they were stored in a –20°C freezer until DNA extraction. All samples were collected in strict accordance with the regulations established by the University of Oklahoma’s Institutional Animal Care and Use Committee (IACUC Permit Nos: R17–019). Field collection and export permits were provided by the Biodiversity Management Bureau (BMB) of the Philippine Department of Environment and Natural Resources (DENR) Nos. 260 (Renewal) and 273 (Renewal).

### DNA Extraction, PCR Amplification, and Sequencing

Genomic DNA was extracted from all 44 swabs using Xpedition^TM^ Soil/Fecal DNA MiniPrep kits (Zymo Research Products). DNA concentration for each extracted sample was determined using a Quantus^TM^ Fluorometer (Promega, Madison, WI, United States). Ten sterile swabs were extracted alongside the 44 samples to be used as negative controls and three of the 10 negative controls were amplified and sequenced using the following methods. Using a one-step Polymerase Chain Reaction (PCR) method, we amplified the V4 hypervariable region of the 16S rRNA gene using primers described in Kozich et al. (2013). Two microliters of PCR product from each sample was visualized using gel electrophoresis and the remaining 18 μL was cleaned with KAPA Pure Beads (Roche Sequencing Solutions, Pleasanton, CA, United States). After quantification, all samples were normalized to 10 nM of DNA before pooling samples into a sterile, 1.5 mL microcentrifuge tube. If the DNA quantity of a sample was above 10 nM, 5 μL of the sample was added to a calculated amount of sterile, laboratory grade water to dilute the sample to 10 nM. After dilution, 4 μL of the diluted PCR product was added to the final pool. In contrast, if the DNA quantity of a sample was below 10 nM, no water was added to the sample and 2 μL of the PCR product was added to the final pool. Sequencing was performed at the University of Oklahoma Consolidated Core Lab using the 2 × 250 bp paired-end sequencing on a single run of an Illumina MiSeq.

### Sequence Analysis

Adapter sequences were trimmed from the paired-end assembled raw sequencing reads using AdapterRemoval v2 (Schubert et al., 2016). Sequence data was analyzed using the QIIME 2 software package (Bolyen et al., 2019), and the sequences were clustered into operational taxonomic units (OTUs) with a closed-reference OTU database at 97% sequencing similarity using VSEARCH (Rognes et al., 2016) against the Silva 132 database (Quast et al., 2012). A total of 9,190 OTUs were found among the 737,795 sequences obtained. The OTU table was rarified to a sequence count of 500 for downstream analyses, which removed 10 samples from the dataset (*N* = 5 each for both mouth and gut samples; Supplementary Table S1 and Supplementary Figure S1). Our decision to rarefy samples to a sequence count of 500 was based on our specific dataset and the associated rarefaction curves (Supplementary Figure S1; Hughes and Hellmann, 2005; Aguirre de Cárcer et al., 2011; Weiss et al., 2017). As a result, the 10 removed samples were excluded from analysis due to low sequence counts and low post-amplification DNA quantities compared to the other samples that amplified well above a sequence count of 500. Raw sequence data generated in this study can be found in the Sequence Read Archive (SRA) under accession no. PRJNA702542. Analysis workflow can be found on Github^1^.

### Statistical Analysis

To evaluate the effect of host ecology and phylogenetic relatedness on gut and mouth diversity, alpha diversity (within sample) and beta diversity (among samples) analyses were performed using QIIME 2. Alpha and beta diversity analyses were performed on both body sites separately and a value of *p* < 0.05 was considered a statistically significant difference. Alpha diversity analyses (Shannon Diversity, Faith’s Phylogenetic Diversity, and Observed OTUs) were performed using QIIME 2 (Bolyen et al., 2019). To test whether the gut microbiomes of these three snake species differed based on host ecology (marine, semi-arboreal, arboreal), we analyzed beta diversity of the gut samples using the phylogeny-based distance matrices Unweighted- and Weighted-Unifrac (Lozupone et al., 2007, 2011) in addition to the Bray-Curtis dissimilarity matrix (Beals, 1984). We performed these three analyses on mouth microbiome samples to test if the microbiomes differed among the three species. Additionally, we separated samples based on the host’s ecology (arboreal, semi-arboreal, marine) and performed the same three analyses on the separated datasets to test for differences in microbial diversity between the two body sites (mouth vs. gut). All beta diversity analyses were visualized by principal coordinate analysis (PCoA) using Qiime 2R (Bisanz, 2018) and Tidyverse (Wickham, 2017) packages in R version 3.6.1 (R Core Team, 2019). Diversity comparisons were conducted using the alpha-group-significance and beta-group-significance plugins in QIIME 2 which performs Pairwise PERMANOVAs to test group significance (Anderson, 2001).

## Results

Our initial microbiome dataset consisted of 22 mouth and 22 gut samples taken from three different venomous snake species (*L. laticaudata*, *T. flavomaculatus*, and *B. dendrophila*) which occupy three distinct habitat types—14 marine samples (*N* = 7 mouth, *N* = 7 gut), 16 semi-arboreal samples (*N* = 8 mouth, *N* = 8 gut), and 14 arboreal samples (*N* = 7 mouth, *N* = 7 gut; Supplementary Table S1). In total, 737,795 sequences were obtained from these 44 samples with a total of 9,190 unique OTUs classified using a 97% sequence similarity threshold against the Silva database (v.132, Quast et al., 2012). After we rarefied the 44 samples to a sequencing depth of 500 for all downstream statistical analyses, 10 out of the 44 samples were removed (mainly *B. dendrophila* samples; Supplementary Table S1). The 34 remaining samples used in subsequent analyses (*N* = 17 mouth, *N* = 17 gut samples; *N* = 13 marine, *N* = 7 semi-arboreal, *N* = 14 arboreal) consisted of 734,366 sequences with 9,033 unique OTUs classified based on 97% similarity using the Silva database (Supplementary Table S1; Quast et al., 2012).

### Taxonomic Composition of GIT Microbial Communities Across Host Species and Ecologies

**FIGURE 1:**
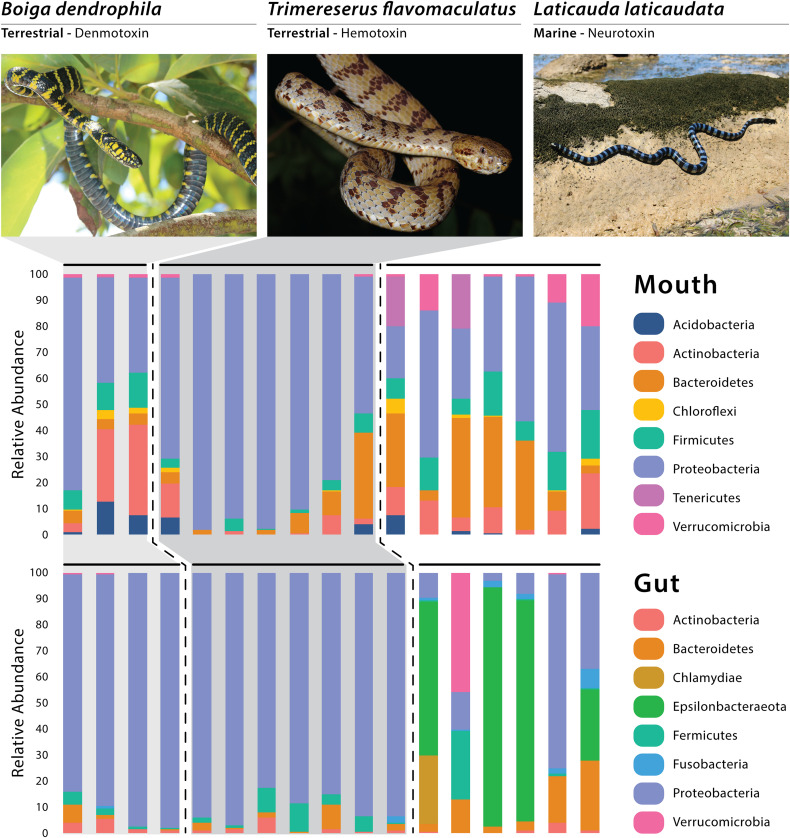
Relative abundance of the dominant bacterial phyla recovered through 16S rRNA amplicon sequencing, with each vertical bar representing an individual swab. Photos of *Boiga* and *Laticauda* by Joseph Brown; Photo of *Trimereserus* by Kai Wang.

A total of four dominant phyla (average relative abundance >1.0%; Suenami et al., 2019) were observed among all gut samples—Proteobacteria (64.87%), Bacteroidetes (5.73%), Firmicutes (4.14%), and Actinobacteria (1.87%; Figure 1). Four bacterial phyla had dominant abundances (avg >1.0%) in the marine gut samples only—Epsilonbacteraeota (43.56%), Verrucomicrobia (7.46%), Chlamydiae (4.40%), and Fusobacteria (2.56%; Figure 1). Among arboreal (*T*. *flavomaculatus*) gut samples only, we found that the most dominant phylum was Proteobacteria (90.15%; Figure 1). Additionally, all seven gut samples collected from this species contained Firmicutes (4.83%), Bacteroidetes (2.47%), and Actinobacteria (1.81%; Figure 1). All other phyla had an average relative abundance below 1%. The same dominant phyla were found among gut samples collected from the semi-arboreal species (*B. dendrophila*). Proteobacteria was also the most dominant phylum within gut samples collected from *B. dendrophila* (92.03%; Figure 1). Additionally, all gut samples collected from this species contained Actinobacteria (3.05%), Bacteroidetes (2.32%), and Firmicutes (2.12%; Figure 1). All other phyla had an average relative abundance below 1%.

All six gut samples from the marine species (*L. laticaudata*) contained Proteobacteria (24.07%), Bacteroidetes (10.94%), Firmicutes (4.61%), Fusobacteria (2.56%), and Actinobacteria (1.25%; Figure 1). Four out of the six gut samples from *L. laticaudata* had high abundances of Epsilonbacteraeota (average for four samples = 65.34%, total average = 43.56%) and two phyla had a high abundance in only one marine gut sample each: Verrucomicrobia (average for one sample = 44.07%) and Chlamydiae (average for one sample = 26.38%; Figure 1).

When evaluating mouth microbiomes only, five dominant phyla were present in samples collected from the three species in varying abundances: Proteobacteria, Bacteroidetes, Firmicutes, Actinobacteria, and Acidobacteria (Supplementary Table S2). Two phyla had especially high relative abundances in two of the seven *L*. *laticaudat*a mouth samples only: Patescibacteria (average for the two samples = 5.06%, total average = 1.59%) and Tenericutes (average for the two samples = 18.5%, total average = 5.33%; Figure 1). Planctomycetes was a dominant phylum in the *B*. *dendrophila* mouth samples only (1.61%; Figure 1). Whereas Verrucomicrobia (*L. laticaudata*: 6.60% and *B*. *dendrophila*: 1.19%) and Chloroflexi (*L. laticaudata*: 1.45% and *B*. *dendrophila*: 1.97%) were dominant phyla among *L. laticaudata* and *B*. *dendrophila* mouth samples but were absent from the *T*. *flavomaculatus* samples (Figure 1).

### Alpha and Beta Diversity Analyses Across Host Ecologies, Species, and Body Site

**FIGURE 2:**
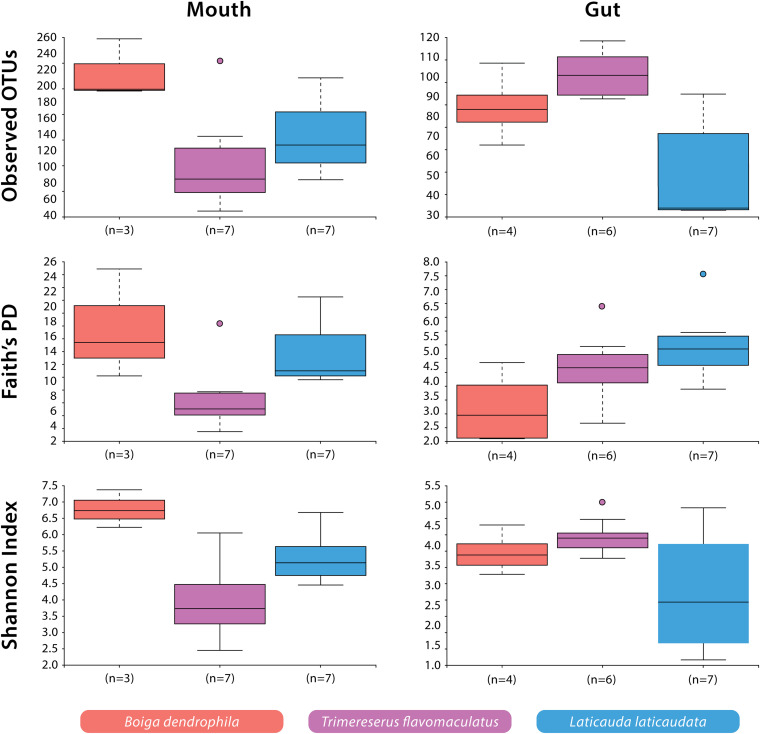
Alpha diversity (Observed OTUs, Faith’s Phylogenetic Diversity, and Shannon Diversity) of bacterial OTUs by host species and body site.

Significant differences were observed among species-specific mouth and gut microbiomes for various alpha diversity metrics evaluated. For mouth microbiome sample comparisons, Shannon Diversity analyses showed significant differences among all three species: *B*. *dendrophila* vs. *T*. *flavomaculatus* (*H* = 5.73; *p*-value = 0.02); *B*. *dendrophila* vs. *L*. *laticaudata* (*H* = 4.69; *p*-value = 0.03); *L*. *laticaudata* vs. *T*. *flavomaculatus* (*H* = 3.92; *p*-value = 0.05; Figure 2). Additionally, Faith’s Phylogenetic diversity analyses supported a significant difference between the mouth microbiome of *T*. *flavomaculatus* and both *L*. *laticaudata* (*H* = 5.59; *p*-value = 0.02) and *B*. *dendrophila* (*H* = 3.75; *p*-value = 0.05), while analysis of observed OTUs showed significant differences between *B*. *dendrophila* and *T*. *flavomaculatus* (*H* = 3.75; *p*-value = 0.05; Figure 2). In comparison, analyses of gut samples supported a significant difference between *L*. *laticaudata* and *T*. *flavomaculatus* (*H* = 7.43; *p*-value = 0.006) in observed OTUs, and between *B*. *dendrophila* and *L*. *laticaudata* (*H* = 4.55; *p*-value = 0.03) for Faith’s Phylogenetic Diversity analyses (Figure 2).

We used Unweighted- and Weighted-Unifrac distance matrices in addition to the Bray-Curtis dissimilarity matrix to analyze beta diversity among mouth and gut microbiome samples. Unweighted-Unifrac analysis resulted in a significant difference when comparing *T*. *flavomaculatus* mouth samples to those collected from *L*. *laticaudata* (pseudo-F = 2.13; *p*-value = 0.011), but no significant difference was found between *B*. *dendrophila* and *T*. *flavomaculatus* (pseudo-F = 1.76; *p*-value = 0.062) and *B*. *dendrophila* and *L*. *laticaudata* mouth samples (pseudo-F = 1.33; *p*-value = 0.131; Supplementary Table S3). Additionally, this analysis found significant differences among gut samples from the semi-arboreal and marine species (pseudo-F = 3.68; *p*-value = 0.004), the semi-arboreal and arboreal species (pseudo-F = 2.09; *p*-value = 0.004), and the arboreal and marine species (pseudo-F = 3.08; *p*-value = 0.001; Supplementary Table S3). When analyzing the habitat types separately, we found significant differences between the body sites of the marine (pseudo-F = 3.39; *p*-value = 0.002) and semi-arboreal species (pseudo-F = 2.89; *p*-value = 0.032), but no significant difference was found between the mouth and gut samples collected from the arboreal species (pseudo-F = 1.39; *p*-value = 0.111; Supplementary Table S3).

Weighted-Unifrac beta diversity analysis of mouth samples revealed significant differences between all three species (*B*. *dendrophila* vs. *L*. *laticaudata*: pseudo-F = 2.47; *p*-value = 0.048; *B*. *dendrophila* vs. *T*. *flavomaculatus*: pseudo-F = 3.92; *p*-value = 0.028; *L*. *laticaudata* vs. *T*. *flavomaculatus*: pseudo-F = 6.60; *p*-value = 0.002; Supplementary Table S3). We found significant differences among gut samples collected from the semi-arboreal and marine species (pseudo-F = 3.83; *p*-value = 0.039) and the arboreal and marine species (pseudo-F = 6.14; *p*-value = 0.003), but not among the semi-arboreal and arboreal species (pseudo-F = 1.11; *p*-value = 0.352; Supplementary Table S3). Among marine samples only, microbiome compositions at the two GIT segments (mouth vs. gut) were significantly different (pseudo-F = 4.41; *p*-value = 0.01), but not among semi-arboreal (pseudo-F = 3.27; *p*-value = 0.122) or arboreal samples (pseudo-F = 0.694; *p*-value = 0.765; Supplementary Table S3).

Analysis of beta diversity using Bray-Curtis dissimilarity matrix yielded significant differences between *B*. *dendrophila* and *L*. *laticaudata* (pseudo-F = 2.18; *p*-value = 0.02), *B*. *dendrophila* and *T*. *flavomaculatus* (pseudo-F = 1.78; *p*-value = 0.034), and *L*. *laticaudata* and *T*. *flavomaculatus* (pseudo-F = 3.71; *p*-value = 0.003; Supplementary Table S3) mouth samples. Additionally, significant differences were found among gut samples collected from the semi-arboreal and marine species (pseudo-F = 5.40; *p*-value = 0.003) and the arboreal and marine species (pseudo-F = 4.88; *p*-value = 0.001), but not among the semi-arboreal and arboreal species (pseudo-F = 1.16; *p*-value = 0.295; Supplementary Table S3). Samples collected from the two body sites (mouth vs. gut) were significantly different among marine samples only (pseudo-F = 3.67; *p*-value = 0.001), but no significant difference between body sites was found among semi-arboreal (pseudo-F = 2.35; *p*-value = 0.169) or arboreal samples (pseudo-F = 0.857; *p*-value = 1.0; Supplementary Table S3).

## Discussion

**FIGURE 3:**
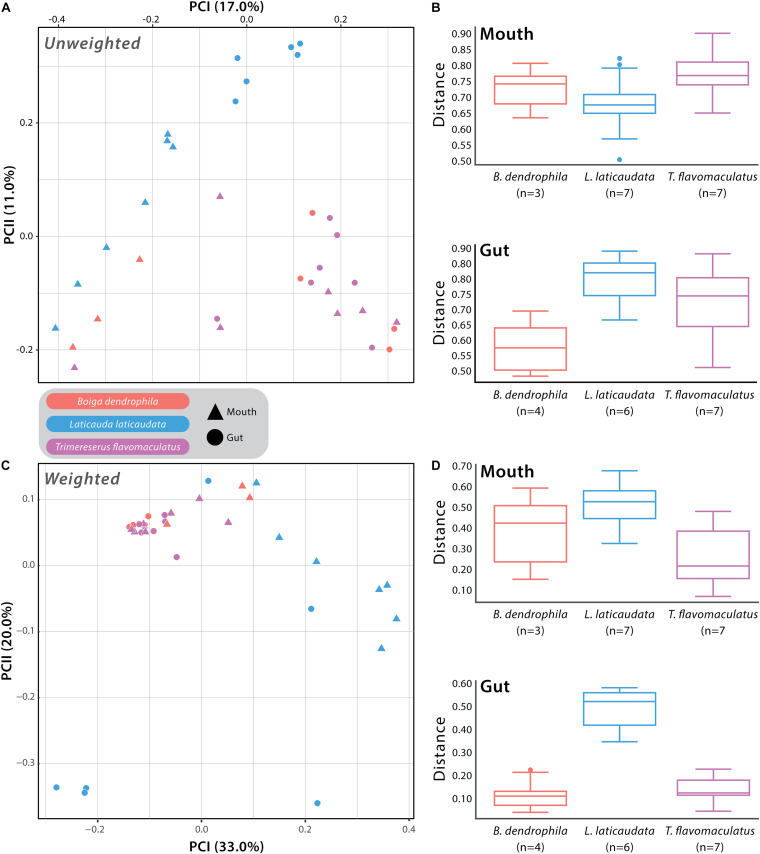
Beta diversity comparisons based on Unweighted- and Weighted-Unifrac distances. **(A)** Principal Coordinates Analysis (PCoA) of all samples with point shape indicating different body sites: triangle = mouth sample and circle = gut sample, and point color indicating the different host species: red = *Boiga dendrophila*, blue = *Laticauda laticaudata*, and purple = *Trimeresurus flavomaculatus*. **(B)** The associated boxplots generated by the PERMANOVA comparing mouth and gut microbiome samples from each host species. **(C)** PCoA of all samples based on Weighted-Unifrac distances with the same point shapes and colors as the Unweighted-Unifrac PCoA **(D)** The associated mouth and gut boxplots generated by the PERMANOVA. Each point represents a single swab. For clarification on sample sizes (n) pre- and post-rarefaction, please see Supplementary Table 1.

In this study, we collected samples from 22 snakes representing three venomous species from the Philippines to investigate whether host ecology and species differences were correlated with potential differences in microbial community structure and diversity within the gut and mouth of the host organisms. The results of this study broaden our understanding of the compositional variation of venomous snake microbiomes at different GIT regions (mouth vs. gut). We found significant differences between the bacterial communities of these GIT body sites in the arboreal, semi-arboreal, and marine species, with these differences being more pronounced in the marine species across all beta diversity analyses (Supplementary Table S3). Additionally, when analyzing both GIT body sites separately, we found mouth microbiome composition and diversity differed between the three host species, revealing that each of the focal species harbored unique mouth microbiomes (Figure 3 and Supplementary Figure S2). Host microhabitat preference (arboreal, semi-arboreal, and marine) was correlated with distinct gut microbial compositions among the three species (Figure 3 and Supplementary Figure S2). The results of this work add to our understanding of regionalized microbiome diversity along the host GIT and establish a foundation for future research to explore the relationship between host-specific microbiomes and snake adaptive evolution.

### Microbial Community Composition Across Host Species and Ecologies

The host species investigated in this study are recognized as members of three distinct snake families (Colubridae: *B. dendrophila*; Elapidae: *L. laticaudata*; Viperidae: *T. flavomaculatus*), providing a unique opportunity to explore interspecific structural differences in the mouth microbiomes of three distantly related venomous snakes (Figure 3 and Supplementary Figure S2). More exciting is that the three species collectively represent two ends of an important ecological spectrum among vertebrates on the planet—marine vs. terrestrial species—with the two terrestrial species occupying distinct microhabitats (arboreal and semi-arboreal). Furthermore, the host species possess distinct dietary preferences that match their broad ecological grouping (marine vs. terrestrial), with the marine species, *Laticauda laticaudata*, recognized as an eel specialist (Tabata et al., 2017), and the two terrestrial snakes, *Trimeresurus flavomaculatus* and *Boiga dendrophila*, are dietary generalists (Creer et al., 2002; Davies and Arbuckle, 2019). Although the current datasets cannot determine whether differences in gut and mouth microbiome composition are due to broader ecological (marine vs. terrestrial), dietary (specialist vs. generalist), taxonomic (phylogenetic relatedness), or other yet unidentified factors, we observed significant host-specific compositional differences in bacterial communities (Figure 3 and Supplementary Figure S2). Additionally, our results reveal important connections to findings in several recent microbiome studies in other vertebrate systems.

Interestingly, gut microbial communities in marine *L. laticaudata* share compositional similarities to microbiomes of loggerhead sea turtles (*Caretta caretta*; Arizza et al., 2019) and American alligators (*Alligator mississippiensis*; Keenan et al., 2013). First, the phylum Epsilonbacteraeota observed recently in gut microbiome samples of loggerhead sea turtles was found in the *L. laticaudata* gut samples only (Figure 1). To date, Epsilonbacteraeota has not been observed in any other terrestrial reptile gut microbiome study (Keenan et al., 2013; Colston et al., 2015; Hyde et al., 2016; Allender et al., 2018; Tang et al., 2019; Zhang et al., 2019). Second, Fusobacteria has been observed as a dominant phylum in the gut microbiomes of American alligators (Keenan et al., 2013) and was observed in *L. laticaudata* gut samples only (Figure 1). Fusobacteria are known to play a role in plaque formation within the oral cavity of mammals (Mira et al., 2004). However, it has been proposed that the phylum may be assisting with digestive organ development and nutrient acquisition in the primarily aquatic American alligators (Keenan et al., 2013).

In comparison, two phyla were observed in mouth samples of *L*. *laticaudata* only: Patescibacteria and Tenericutes (Figure 1). We have not encountered a study that includes Patescibacteria as a dominant phylum in the reptilian mouth or gut microbiomes, but Tenericutes has been observed in the upper GIT (stomach and esophagus) of the red-necked keelback, *Rhabdophis subminiatus*, a semi-aquatic snake (Tang et al., 2019), the stomach of the giant African snail (*Achatina fulica*; Pawar et al., 2012), the oral and fecal microbiota of a passerine bird (*Parus major*; Kropáčková et al., 2017), and in the gut microbiomes of numerous species of fish (Sullam et al., 2015; Llewellyn et al., 2016). Tenericutes are also dominant members of the coral microbiome (Kellogg et al., 2009; Gray et al., 2011), which adds support for the phylum’s connection to aquatic, and particularly marine, organisms (Colston and Jackson, 2016).

In addition to the observed similarities in microbial community composition among aquatic reptiles, both mouth and gut samples of the terrestrial snakes (*T. flavomaculatus*, *B. dendrophila*) showed interesting compositional patterns, with both species dominated by Proteobacteria (Figure 1), a phylum observed at much lower relative abundance in most *L. laticaudata* marine samples, particularly gut samples (Figure 1). Additionally, one dominant phylum, Planctomycetes, was observed exclusively in the mouth samples of *B*. *dendrophila* (Figure 1) but has also been observed previously in small relative abundances associated with the GIT of red-necked keelback snakes (*Rhabdophis subminiatus*; Tang et al., 2019), Galápagos land iguanas (*Conolophus subscristatus*; Hong et al., 2011), and Santa Fe land iguanas (*C*. *pallidus*; Hong et al., 2011). Together these findings reveal new insights into bacterial phyla that may be associated with reptiles that occupy marine vs. terrestrial habitats differentially, presenting exciting directions for future research.

Finally, with the three focal snakes each possessing distinct toxins in their venom (neurotoxin, hemotoxin, and denmotoxin; Supplementary Table S1), we were interested in how our findings might relate to microbiome work in other venomous taxa. Interestingly, members of the phylum Chloroflexi were present only in the *L*. *laticaudata* and *B. dendrophila* mouth samples and have been found in the oral cavity of several other snake species, including the Indian cobra and king cobra, which like *L. laticaudata*, are both members of the snake family Elapidae (Krishnankutty et al., 2018). Given the growing interest in venomics and improving our understanding of venom evolution and antimicrobial resistance, it is critical that baseline datasets be established for the identification and understanding of host-specific microbiome composition present in venom microenvironments (Conlin et al., 2014; Adnani et al., 2017; Esmaeilishirazifard et al., 2018; Krishnankutty et al., 2018). Our findings add to this small but growing body of literature with the hope that future studies will be capable of testing for evidence of coevolutionary processes between mouth and venom gland microbiomes and the evolution of diverse venom systems in snakes.

### Comparisons to the Culture-Dependent Snake Microbiome Literature

Although we utilized high-throughput sequencing methods for our study, similarities can be found with several wild snake microbiome studies previously conducted using culture-dependent techniques (Shek et al., 2009; Lam et al., 2011; Dehghani et al., 2016; Lukač et al., 2017). Lukač et al. (2017) sampled the oral cavity and cloaca of wild four-lined snakes (*Elaphe quatuorlineata*) and reported several genera of pathogenic bacteria that were also observed in mouth and/or gut samples in this study. First, *T. flavomaculatus* gut samples revealed prominent relative abundances of *Bacillus*, *Escherichia coli*/*Shigella* spp., *Pseudomonas*, *Serratia*, and *Stenotrophomonas*. Additionally, among mouth samples analyzed in our study, the presence and relative abundances of two potentially pathogenic bacterial genera stood out: (1) *Escherichia coli*/*Shigella* spp. were found in high relative abundances among mouth samples from all three host species we surveyed (average relative abundances = *B*. *dendrophila*: 10.46%; *L*. *laticaudata*: 2.81%; *T*. *flavomaculatus*: 25.73%); (2) One *B*. *dendrophila* mouth sample had a high relative abundance of *Aeromonas* (56.94%), which is a bacterium known to cause snakebite wound infections (Lam et al., 2011).

Two other culture-dependent studies sampled the oral cavity of snakes collected from the same location in Hong Kong (Shek et al., 2009; Lam et al., 2011), also belonging to the same families as the snakes sampled here (Colubridae, Elapidae, and Viperidae; Lam et al., 2011). Snakes sampled by Shek et al. (2009) and Lam et al. (2011) had *Providencia rettgeri* within their oral cavities, but the genus was absent from snakes sampled by Lukač et al. (2017). Comparatively, the bacterial genus *Providencia* was observed in high relative abundances (i.e., average relative abundance 79.69% in *T. flavomaculatus*) among mouth samples from each focal host species in our study. Finally, two species of the bacterial genus *Chryseobacterium* have been observed in the oral cavities of the Chinese cobra (*Naja atra*) and bamboo pit viper (*Trimeresurus albolabris*; Shek et al., 2009), and the genus was also found among *T*. *flavomaculatus* mouth samples from our study (average relative abundance = 6.15%). The presence of *Chryseobacterium* and other potentially pathogenic bacteria observed in our study may indicate that certain host individuals of the three focal snakes may harbor disease-causing bacteria within their mouths and guts. This is particularly true for *T*. *flavomaculatus*, which tended to have higher relative abundances for bacterial genera shown to be potentially pathogenic within culture-dependent studies conducted to date (Shek et al., 2009; Lam et al., 2011; Lukač et al., 2017). The implications of this extend to wildlife trade, where unmanaged and/or illegal trade of wildlife can have negative impacts on human health, including zoonotic disease transmission, at times through the consumption of wild meats (i.e., Covid-19 and HIV-1; Roe, 2008).

## Conclusion

Despite gradual improvements to our understanding of microbial differences between distinct body sites across the reptilian GIT (Keenan et al., 2013; Colston et al., 2015; Hyde et al., 2016; Tang et al., 2019), there remains a fundamental paucity of information on the composition, diversity, and functional capabilities of snake microbiomes (Krishnankutty et al., 2018). However, developing a baseline understanding of venomous snake microbiomes with regional associations in the body (e.g., GIT vs. venom gland) will be vital in order to address the important roles host-specific microbiomes may have played in venomous snake adaptive evolution. Additionally, although the use of 16S rRNA gene amplicon sequencing is often the first step to elucidating important patterns on compositional differences among host-associated bacterial communities, other genomic sequencing technologies (i.e., shotgun metagenomic sequencing) present exciting opportunities to begin identifying potential functional roles of these microbiomes and how they interact with their hosts (Rebollar et al., 2018). With the results of this comparative study of three different host species, each representing a unique snake family (Colubridae, Elapidae, Viperidae), we can continue to address this information gap for one of the most ecologically diverse and speciose vertebrate groups on the planet.

## Data Availability Statement

The datasets presented in this study can be found in online repositories. The names of the repository/repositories and accession number(s) can be found below: NCBI SRA; PRJNA702542.

## Ethics Statement

The animal study was reviewed and approved by the University of Oklahoma’s Institutional Animal Care and Use Committee.

## Author Contributions

SS and CS contributed to the study conception and design with advisement from TC. CS secured funding for the field expedition, sample collection, extraction, amplification, and 16S rRNA sequencing. Field surveys in the Philippines were conducted by SS and CS. DNA extraction, amplification, and sequencing led by SS. Data analyses were performed by SS with assistance from TC. The first draft of the manuscript was written by SS, with contributions and reviews by CS and TC. All authors read and approved the final version of the manuscript.

## Conflict of Interest

The authors declare that the research was conducted in the absence of any commercial or financial relationships that could be construed as a potential conflict of interest.

## Footnotes

1 https://github.com/sierra-smith/16S_Microbiome_pipeline.git
