# Supplementary material for: Venomous Snakes Reveal Ecological and Phylogenetic Factors Influencing Variation in Gut and Oral Microbiomes
Source: Front Microbiol. 2021 Mar 26;12:657754. doi: 10.3389/fmicb.2021.657754 (PMC8032887; doi:10.3389/fmicb.2021.657754)
Supplement: Supplementary file 1 [file Data_Sheet_1.DOCX]

Supplementary Material

## Supplementary Figure Legends

**Supplementary Figure 1.** Alpha diversity rarefaction curves based on Shannon diversity. Each curve is representative of a single swab. Based on these curves, we decided to rarefy all samples to a sequencing depth of 500 sequences.

**Supplementary Figure 2.** Beta diversity analysis based on the Bray-Curtis dissimilarity matrix. (A) PCoA of all samples with the different body sites represented as triangles = mouth samples and circle = gut samples, and point color representing the different host species: red = *Boiga dendrophila*, blue = *Laticauda laticaudata*, and purple = *Trimeresurus flavomaculatus*. (B) Boxplots generated by the PERMANOVA comparing the mouth and gut samples from each host species.

**Supplementary Table Legends**

**Supplementary Table 1.** Sample sizes for the total number of mouth and gut samples before and after rarefaction to exclude samples with low sequence number (i.e. <500 reads per sample).

**Supplementary Table 2.** Relative abundances of the five dominant (relative abundance > 1%) microbial phyla present in all mouth samples from the three host species.

**Supplementary Table 3.** Beta diversity results for each analysis (Unweighted-Unifrac, Weighted-Unifrac, and Bray-Curtis).
