# Supplementary figures and images for: Venomous Snakes Reveal Ecological and Phylogenetic Factors Influencing Variation in Gut and Oral Microbiomes
Source: Front Microbiol. 2021 Mar 26;12:657754. doi: 10.3389/fmicb.2021.657754 (PMC8032887; doi:10.3389/fmicb.2021.657754)

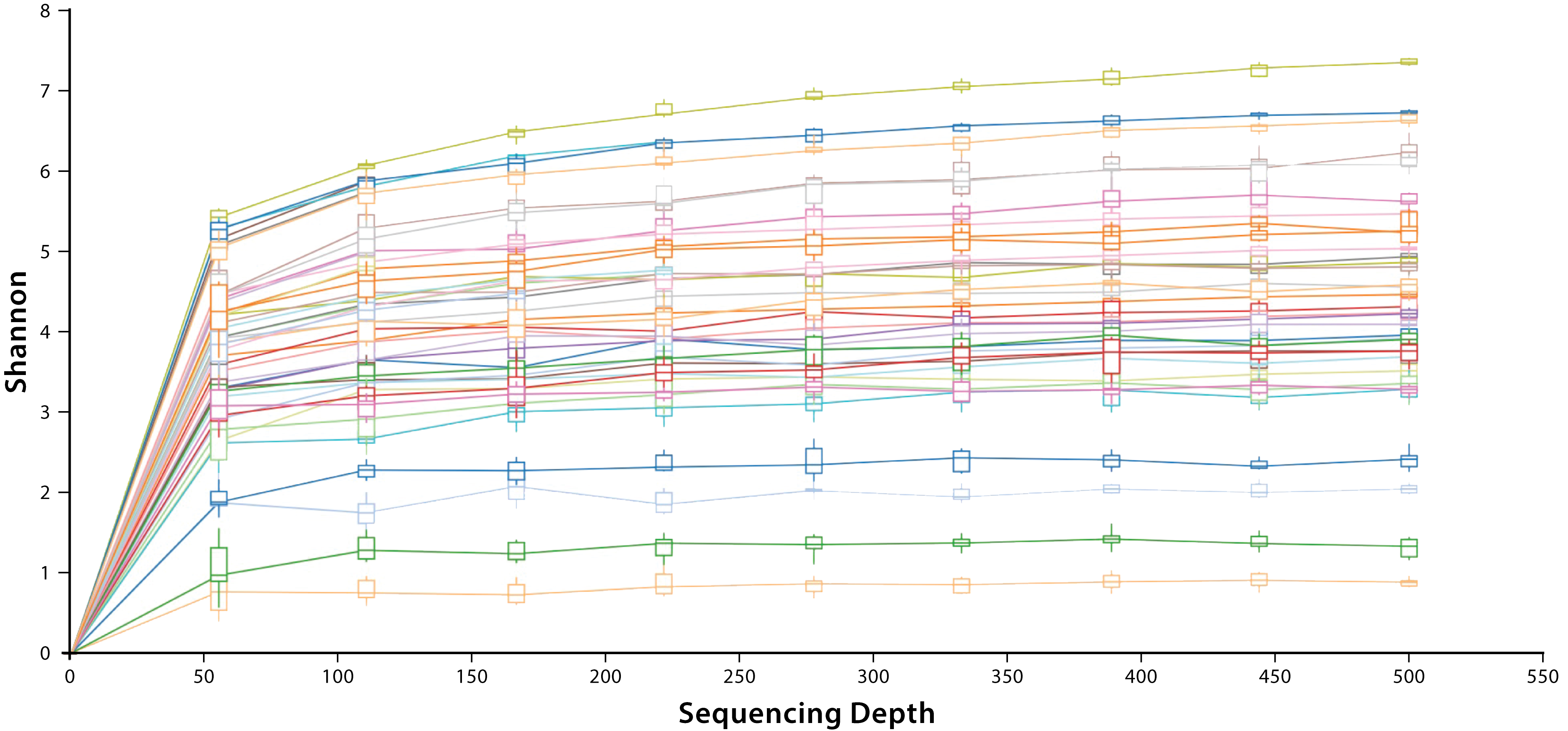

Supplement: Supplementary file 2 [file Image_1.TIF]

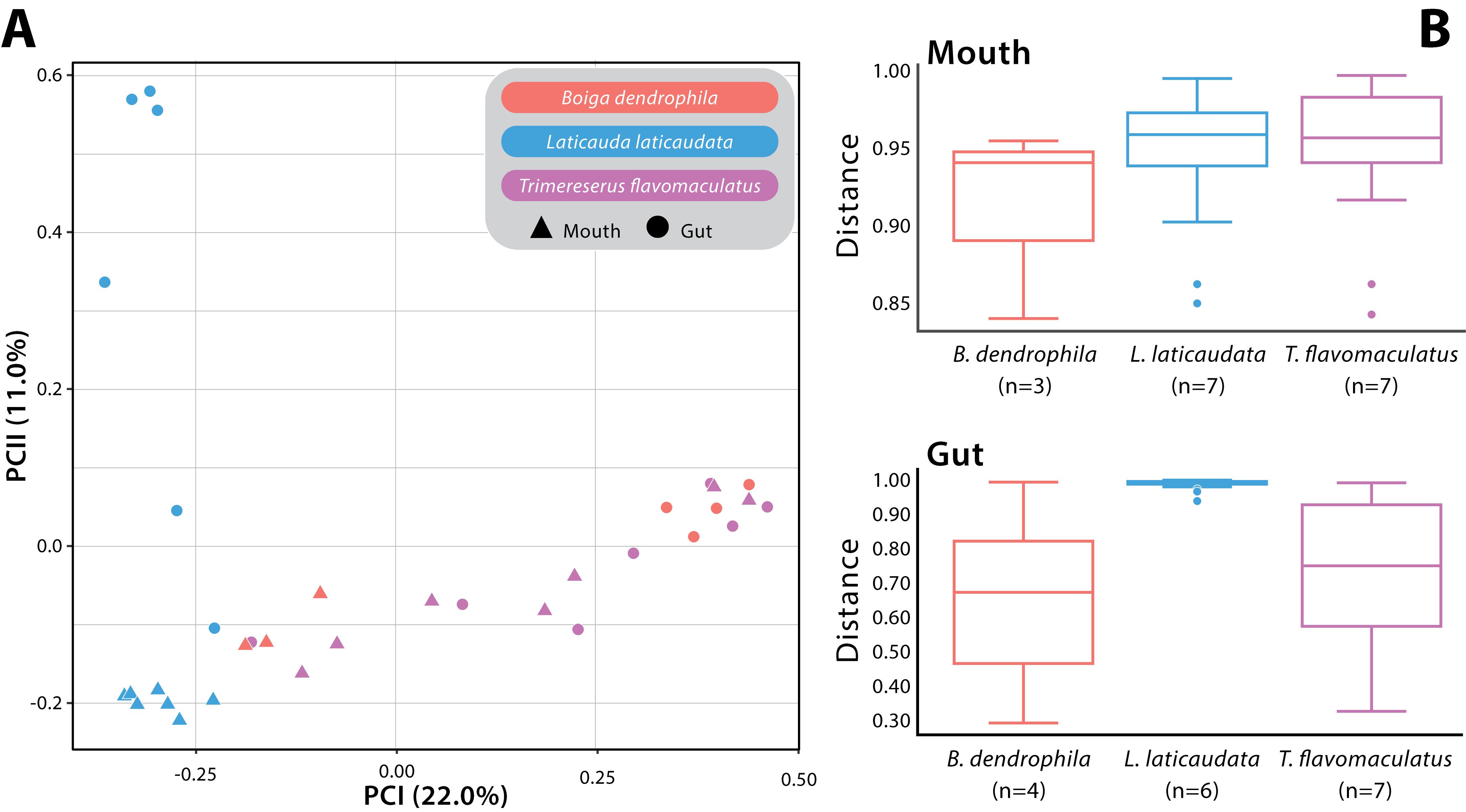

Supplement: Supplementary file 3 [file Image_2.TIF]
